# Immune checkpoint inhibitor-associated uveitis in a patient with breast cancer and a remote history of Vogt–Koyanagi–Harada disease: a case report

**DOI:** 10.1007/s13691-026-00873-3

**Published:** 2026-05-08

**Authors:** Konoka Uraguchi, Kanako Saito, Kumiko Kato, Rena Yamakado, Akira Tsunoda, Hiroki Oka, Yasutaka Tono, Kosuke Kawaguchi, Isao Tawara, Toshiro Mizuno

**Affiliations:** 1 Department of Medical Oncology, Mie University Hospital, 2-174, Edobashi, Mie, 514-8507 Tsu, Japan; 2 Department of Ophthalmology, Mie University Graduate School of Medicine, Tsu, Japan; 3 Department of Breast Center, Mie University Hospital, Tsu, Japan; 4 Department of Hematology and Oncology, Mie Graduate School of Medicine, Tsu, Japan

**Keywords:** Breast cancer, Uveitis, Pembrolizumab, irAE, Autoimmune disease

## Abstract

Immune checkpoint inhibitors (ICIs) have demonstrated substantial efficacy in the treatment of triple-negative breast cancer (TNBC). However, appropriate management of immune-related adverse events (irAEs) remains essential, and patients with a history of autoimmune disease have an increased risk of developing irAEs. Immune-related uveitis represents an uncommon irAE overall and has been reported most frequently in melanoma, whereas reports among patients with BC remain rare. We describe a case of ICI-associated uveitis occurring shortly after treatment initiation in a patient with a very remote history of autoimmune uveitis. A woman in her 50s with locally advanced TNBC (cT4bN0M0, stage IIIB) and a history of Vogt–Koyanagi–Harada disease–associated uveitis in complete remission for more than 25 years received neoadjuvant chemotherapy combined with pembrolizumab. Three weeks after initiation, decreased visual acuity developed in the left eye, and grade 2 uveitis was diagnosed as an irAE. Pembrolizumab was discontinued, and systemic corticosteroid therapy was initiated, resulting in early ophthalmic improvement. Subsequently, Stevens–Johnson syndrome developed after prophylactic trimethoprim–sulfamethoxazole administration, necessitating steroid pulse therapy and high-dose intravenous immunoglobulin, after which the uveitis completely resolved. Neoadjuvant chemotherapy was continued without pembrolizumab, followed by mastectomy, which revealed ypT1aN0 disease. At 2 years of follow-up, the patient remains disease-free without recurrence of uveitis. This case indicates that a remote history of autoimmune uveitis may represent a risk factor for ICI-associated uveitis, supporting the need for careful pretreatment assessment, vigilant monitoring, and close collaboration with ophthalmologists.

## Introduction

Immune checkpoint inhibitors (ICIs) are widely used across various cancer types and have significantly improved treatment outcomes. However, by enhancing immune activation, ICIs can induce immune-related adverse events (irAEs), which present as autoimmune-like toxicities [1]. Consequently, the administration of ICIs in patients with pre-existing autoimmune diseases raises concerns regarding an increased risk of irAEs, including the potential reactivation or recurrence of underlying autoimmune conditions [2, 3].

IrAEs can affect multiple organ systems, including the skin, gastrointestinal tract, lungs, endocrine organs, and eyes, and demonstrate a broad spectrum of clinical manifestations [1]. Uveitis represents an uncommon irAE, with an estimated incidence ranging from 0.3% to 6% [4]. Despite this low frequency, uveitis constitutes the most frequently reported ocular irAE and carries clinical significance because severe or inadequately treated disease may result in vision-threatening complications [5]. Uveitis is broadly classified into infectious and non-infectious forms, with the latter being immune-mediated and associated with systemic inflammatory diseases such as Vogt–Koyanagi–Harada (VKH) disease, sarcoidosis, and Behçet’s disease. In particular, immune cross-reactivity against melanocyte-associated antigens in melanin-containing ocular tissues, including the uvea and choroid, has been proposed as a mechanism of ICI-associated uveitis. Such ocular inflammation has been reported most commonly in melanoma, often presenting as VKH-like disease, whereas occurrence among patients with breast cancer (BC) remains exceedingly rare [6–8]. This report describes a case of uveitis that developed during ICI-based therapy in a patient with BC and a remote history of VKH disease, highlighting a potential risk of ICI-associated uveitis in patients with prior autoimmune uveitis despite long-term disease quiescence.

## Case report

A woman in her 50s presented with a mass in the right breast. As the lesion progressively enlarged, evaluation occurred at a local hospital, where BC was diagnosed, followed by referral to our institution. Physical examination identified a hard mass measuring approximately 9 cm in the right breast, accompanied by erythema and skin thickening. Imaging studies demonstrated a 72-mm mass in the right breast and slight enlargement of the right axillary lymph nodes, without evidence of distant metastasis. Core needle biopsy of the breast mass confirmed invasive ductal carcinoma of the triple-negative (TN) subtype, with negative estrogen receptor (ER), progesterone receptor (PgR), and human epidermal growth factor receptor 2 (HER2) expression. Cytological examination of the right axillary lymph node was negative for malignancy. The clinical stage was cT4bN0M0, corresponding to stage IIIB disease.

The patient was scheduled to receive neoadjuvant chemotherapy according to the KEYNOTE-522 regimen [9]. During the neoadjuvant phase, pembrolizumab (an anti–programmed death-1 antibody) was scheduled to be administered at 200 mg every 3 weeks for up to eight cycles. Concurrently, weekly paclitaxel (80 mg/m^2^) was planned for 12 doses, with carboplatin administered either weekly at an AUC of 1.5 or every 3 weeks at an AUC of 5.0, followed by either doxorubicin (60 mg/m^2^) or epirubicin (90 mg/m^2^), each in combination with cyclophosphamide (600 mg/m^2^), every 3 weeks for four cycles. In the adjuvant phase, pembrolizumab was scheduled to be administered at either 200 mg every 3 weeks for nine cycles or 400 mg every 6 weeks for five cycles.

Her Eastern Cooperative Oncology Group (ECOG) performance status was 0. The patient had a history of VKH disease at 25 years of age without extraocular manifestations such as auditory symptoms, cutaneous findings, or neurological signs. She was treated with steroid pulse therapy, and the uveitis resolved completely without relapse after cessation of steroid treatment. She had no other past medical history. Because she had no ocular symptoms at the time of her breast cancer diagnosis, she did not undergo ophthalmologic evaluation before pembrolizumab treatment. Baseline laboratory testing demonstrated elevated anti-thyroid peroxidase antibody (467 IU/mL) and anti-thyroglobulin antibody (1,080 IU/mL) levels in the absence of a prior history of thyroid disease, with no other clinically significant abnormalities.

**Fig. 1:**
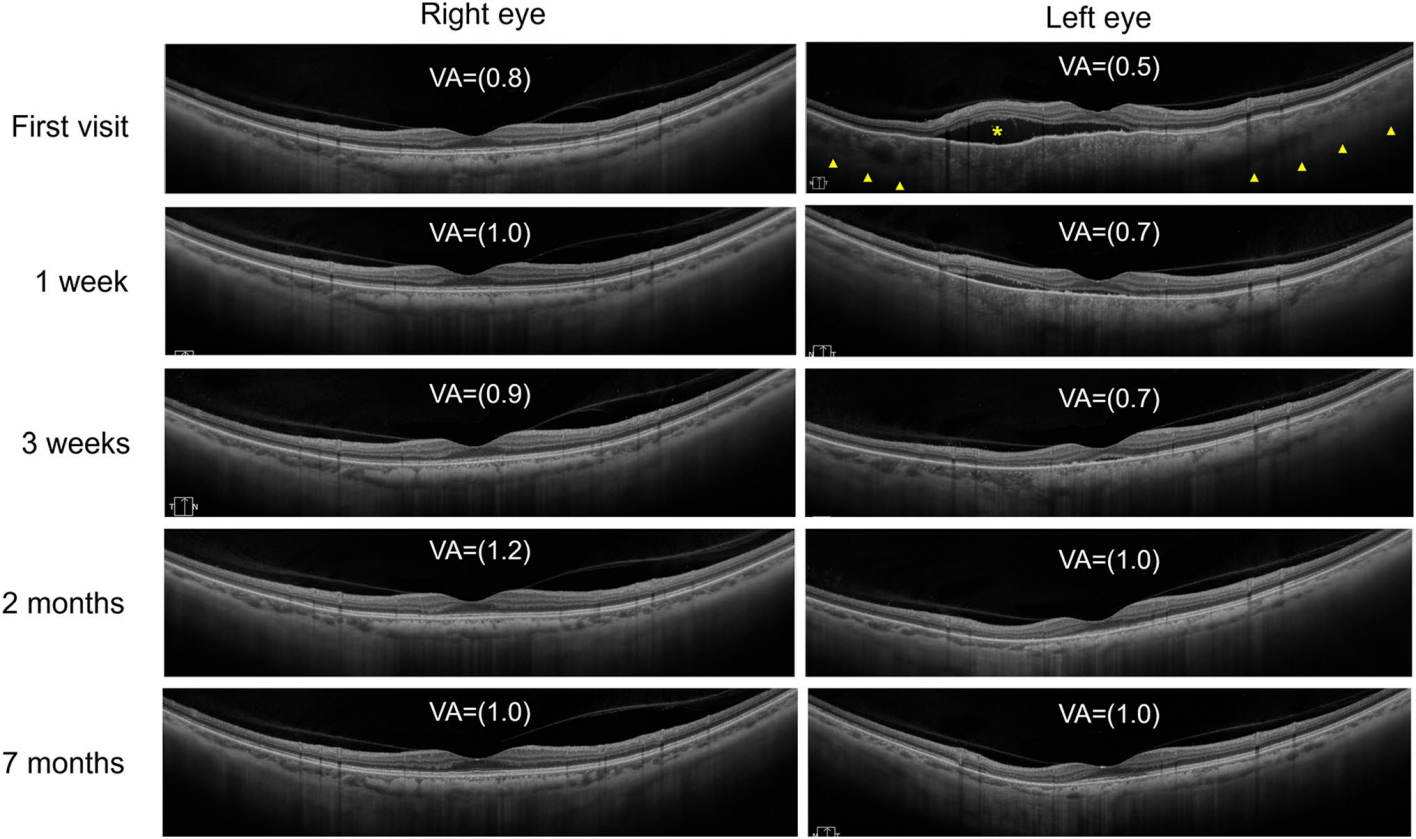
Bilateral optical coherence tomography (OCT) images. At the initial visit, serous retinal detachment (yellow asterisk) and choroidal thickening (yellow arrowheads) were observed in the left eye, and both findings improved rapidly after treatment. No definite structural abnormalities were detected in the right eye on OCT. Best-corrected visual acuity (VA) gradually recovered during follow-up

**Fig. 2:**
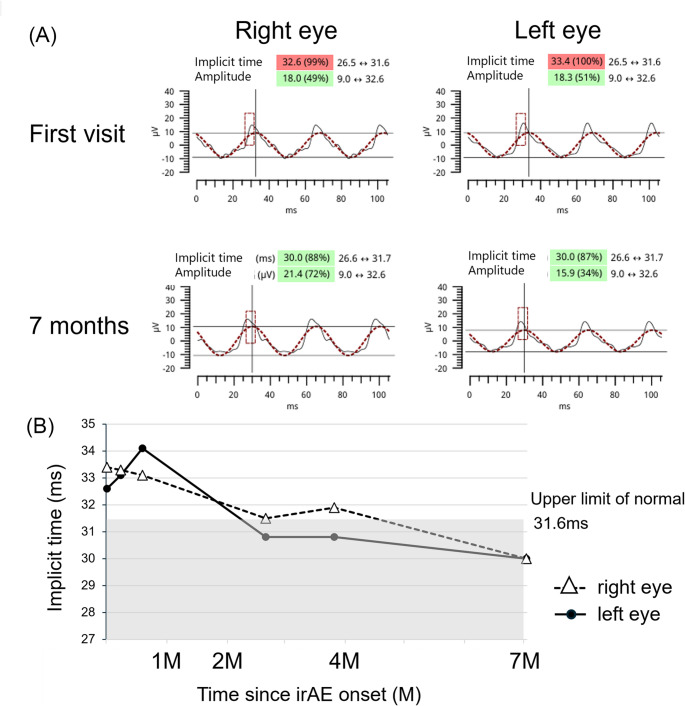
Flicker electroretinography (ERG) findings. (**A**) Bilateral full-field 28.3-Hz flicker ERG recording obtained using skin electrodes. At presentation, implicit times were prolonged in both eyes (right, 32.6 ms; left, 33.4 ms), indicating bilateral retinal dysfunction. At 7 months, implicit times improved to within the normal range in both eyes (30.0 ms). (**B**) Time course of flicker ERG implicit times. The gray shaded area indicates the normal reference range (≤ 31.6 ms). Implicit times gradually improved after initiation of treatment and remained within the normal range from 3 months onward

Twenty days after the first administration of pembrolizumab in combination with chemotherapy, blurred vision developed in the left eye. As the symptom was very mild on day 22 (Cycle 2, day 1), the second dose of pembrolizumab was administered as scheduled. However, the ocular symptoms worsened 1 week later, prompting referral to an ophthalmologist. On ophthalmologic examination, decimal best-corrected visual acuity (BCVA) was 0.8 in the right eye and 0.5 in the left eye. Optical coherence tomography (OCT) revealed serous retinal detachment and choroidal thickening in the left eye (Fig. 1), whereas no definite structural abnormality was detected in the right eye. In contrast, full-field 28.3-Hz flicker electroretinography (ERG) demonstrated prolonged implicit times in both eyes, indicating bilateral retinal dysfunction (Fig. 2A, B). These findings supported a diagnosis of grade 2 uveitis as an irAE induced by pembrolizumab.

Pembrolizumab was discontinued, and oral prednisolone was initiated at a dose of 20 mg/day. Within 1 week, OCT demonstrated a reduction in subretinal fluidin the left eye, and BCVA improved to 1.0 in the right eye and 0.7 in the left eye (Fig. 1). Pembrolizumab was not resumed because of concern regarding reactivation of uveitis. As the uveitis did not worsen under low-dose steroid therapy, weekly paclitaxel and carboplatin were continued without interruption. In the context of ongoing systemic corticosteroid therapy, prophylactic trimethoprim–sulfamethoxazole (TMP-SMX) was initiated. However, 10 days later, the patient developed a generalized erythematous rash with high fever and mucosal involvement, leading to a diagnosis of Stevens–Johnson syndrome (SJS). Treatment consisted of steroid pulse therapy and high-dose intravenous immunoglobulin, and all anticancer treatments were temporarily discontinued for safety.

**Fig. 3:**
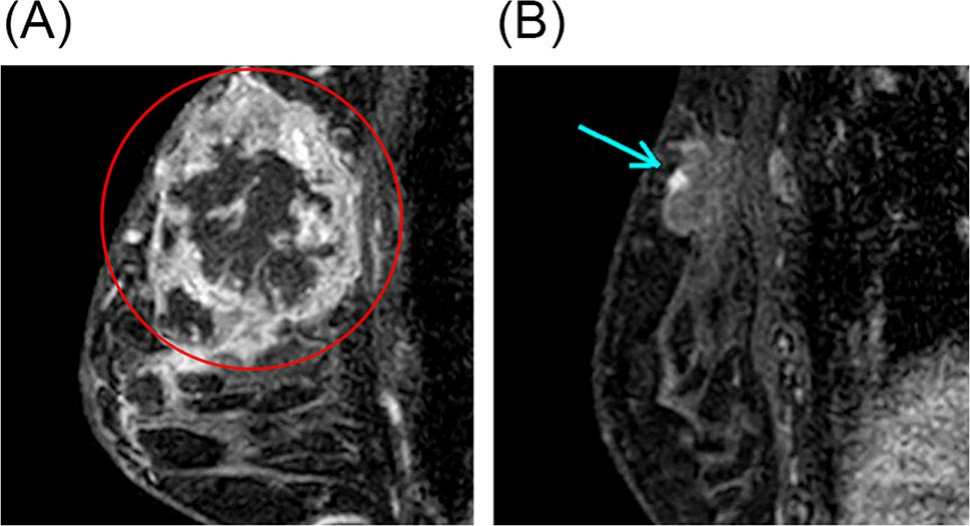
Contrast-enhanced magnetic resonance imaging of the right breast. (**A**) Before treatment. (**B**) After neoadjuvant chemotherapy

After resolution of the cutaneous lesions, prednisolone was gradually tapered, and neoadjuvant chemotherapy with AC (doxorubicin and cyclophosphamide) was resumed without pembrolizumab. The subretinal fluid resolved completely after completion of steroid therapy for SJS (Fig. 1, fourth row) and BCVA improved to 1.2 in the right eye and 1.0 in the left eye. Flicker ERG implicit times also gradually returned to within the normal range (Fig. 2A, B). During AC therapy, hypothyroidism developed as an irAE despite discontinuation of pembrolizumab. After completion of neoadjuvant chemotherapy, marked regression of the primary tumor was observed, followed by mastectomy with sentinel lymph node biopsy (Fig. 3). Histopathological examination revealed residual invasive ductal carcinoma, classified as ypT1aN0, corresponding to ypStage I disease, with a histological treatment response of grade 2b. Postoperative management included radiotherapy to the chest wall, followed by 6 months of adjuvant capecitabine (1250 mg/m^2^, day 1–14, q3w, 8 cycles) in place of pembrolizumab.

At 2 years after surgery, the patient remains alive without recurrence of BC and without recurrence of uveitis.

**Fig. 4:**
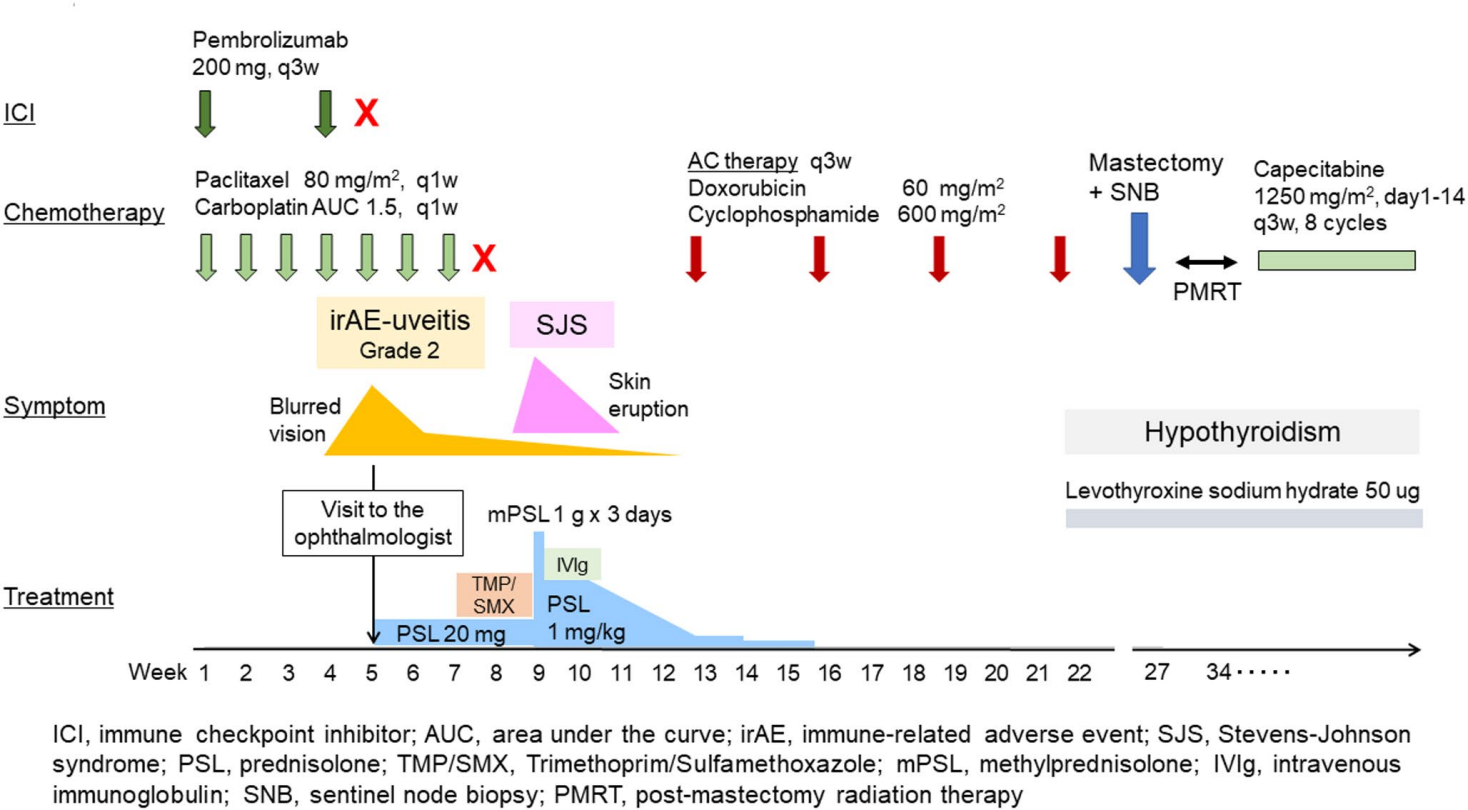
The treatment course of the present case

The treatment course of the present case is shown in Fig. 4.

## Discussion

This case report describes a patient with a remote history of uveitis who developed irAE–associated uveitis following ICI-based therapy for BC, occurring 25 years after the initial episode.

The reported incidence of irAE-associated uveitis ranges from 0.3% to 6% [4], with melanoma representing the most common underlying malignancy, followed by lung cancer and urological cancers [5, 10]. A retrospective cohort study demonstrated that the 1-year incidence of uveitis among patients treated with ICIs was significantly higher among individuals with melanoma compared to non-melanoma malignancies (1.2% vs. 0.2%), with an adjusted odds ratio of 6.45 [6]. Similarly, a recent systematic review including 52 patients reported melanoma in 69.2% of irAE uveitis cases, whereas renal cell carcinoma and lung cancer each accounted for 11.5% [8]. One proposed mechanism underlying the high incidence of irAE uveitis in melanoma involves immune cross-reactivity between melanoma antigens and melanocytes within the retina and choroid, potentially leading to VKH-like pathology [11]. Additionally, VKH disease has been reported more frequently among individuals with specific human leukocyte antigen (HLA) genotypes [11, 12]. Regarding ICI regimens, 63.5% of patients who developed irAE uveitis received programmed death-1 (PD-1) inhibitor monotherapy, whereas 19.2% received combination therapy that included a cytotoxic T-lymphocyte–associated antigen 4 (CTLA-4) inhibitor [8]. Dual PD-1 and CTLA-4 blockade has been used more frequently in melanoma, renal cell carcinoma, and lung cancer, a pattern that may partially explain the higher incidence of irAE uveitis reported in these malignancies.

In contrast, irAE-associated uveitis is rarely reported among patients receiving ICIs for BC. In the KEYNOTE-522 trial, which employed the regimen administered in the present case, uveitis occurred in only two of 784 patients (0.3%) [9]. Similarly, the KEYNOTE-355 trial involving patients with metastatic TNBC treated with pembrolizumab plus chemotherapy reported a uveitis incidence of 0.2% [13]. Other pivotal clinical trials evaluating ICI-based regimens for early-stage or advanced BC either did not report uveitis or reported very low frequencies [14–20] (Table 1). The relatively low frequency of irAE uveitis in breast cancer may be explained, at least in part, by the absence of melanocytes in normal breast tissue. However, because melanocytes are likewise rarely identified in most other non-melanoma tumors treated with ICIs, the greater number of reported cases in lung cancer and renal cell carcinoma may be better explained by differences in ICI responsiveness, the intensity of immune activation, earlier and more widespread clinical implementation of ICIs, and the use of anti–PD-1/anti–CTLA-4 combination regimens, rather than by organ-specific biological features. Given the rarity of this toxicity, however, such interpretations should be considered hypothesis-generating.

**Table 1:** Incidence of uveitis or ocular inflammatory events reported in pivotal clinical studies using ICIs in patients with breast cancer

Agent	Target	Clinical trial	Patient population	Treatment regimen (cytotoxic agents)	Reported incidence of uveitis (ocular inflammatory events)	Number of patients	Reference
Pembrolizumab	PD-1	KEYNOTE-522	EBC, TNBC	Paclitaxel + carboplatin → anthracycline/cyclophosphamide	0.3%	2/781	[9]
Atezolizumab	PD-L1	IMpassion031	EBC, TNBC	Nab-paclitaxel → anthracycline/cyclophosphamide	1% (ocular inflammatory toxicity)	2/164	[14]
Nivolumab	PD-1	CheckMate7FL	EBC, ER+/HER2-	Anthracycline/taxane-based chemotherapy	Not reported	? /262	[15]
Nivolumab + Ipilimumab	PD-1 + CTLA-4	Adaptive trial (Phase II)	EBC, TNBC	Carboplatin + docetaxel	None	0/46	[16]
Atezolizumab	PD-L1	ALEXANDRA / IMpassion030	EBC, TNBC	Anthracycline/taxane-based chemotherapy (adjuvant)	0.64%	7/1,093	[17]
Atezolizumab	PD-L1	IMpassion130	MBC, TNBC	Nab-paclitaxel	< 1% (immune-related ocular inflammation)	? /452	[18]
Atezolizumab	PD-L1	IMpassion131	MBC, TNBC	Paclitaxel	0.9% (ocular inflammatory toxicity)	4/432	[19]
Pembrolizumab	PD-1	KEYNOTE-355	MBC, TNBC	Investigator’s choice chemotherapy	0.2%	1/596	[﻿^13^] 16 September 2021 EMA/CHMP/563896/2021
Pembrolizumab	PD-1	KEYNOTE-119	MBC, TNBC	Investigator’s choice chemotherapy	Not reported	? /309	[20]

“None” indicates that no uveitis or ocular inflammatory events were reported, whereas “Not reported” indicates that such events were not specifically described in the published safety data

The number of patients refers to those included in the safety analysis relevant to ocular adverse events

EBC, early breast cancer; TNBC, triple-negative breast cancer; ER, estrogen receptor; HER2, human epidermal growth factor-2; MBC, metastatic breast cancer; PD-1, programmed cell death protein 1; CTLA-4, cytotoxic T-lymphocyte-associated antigen 4; ICIs, immune checkpoint inhibitors

Patients with pre-existing autoimmune diseases have an increased risk of developing irAEs, including flares of underlying conditions. A meta-analysis including 23,897 patients with cancer and pre-existing autoimmune diseases reported an overall irAE incidence of 61% after ICI-based therapy, with 36% representing flares of the underlying autoimmune disease [3]. Another study demonstrated an association between pre-existing autoimmune disease and irAE development, with an adjusted odds ratio of 2.52 [2]. These findings emphasize the importance of obtaining a detailed medical history and performing careful risk assessment before initiating ICI-based therapy.

In the present case, the distinction between a flare of VKH disease and ICI-associated VKH-like uveitis is inherently challenging. However, several features favored ICI-associated uveitis in our patient: the onset occurred shortly after pembrolizumab initiation, the patient had remained free of recurrence for more than 25 years, and the ocular inflammation responded promptly to discontinuation of pembrolizumab and relatively low-dose systemic corticosteroid therapy. In contrast, recurrent VKH generally requires more intensive and prolonged immunosuppressive treatment to achieve sustained disease control. Therefore, although recurrence of VKH cannot be completely excluded, the clinical course in this case was considered to be more consistent with ICI-associated uveitis.

Large cohort studies have demonstrated that patients with a prior history of uveitis are at increased risk of ophthalmic irAE, including uveitis, after ICI-based therapy. One study reported recurrence rates of 44% (44/100) after pembrolizumab administration and 50% (8/16) after ipilimumab administration [7]. Another report demonstrated 1-year recurrence rates of 28.6% among patients with melanoma and 12.6% among patients with non-melanoma malignancies who had a history of uveitis after ICI initiation, supporting pre-existing uveitis as a risk factor for ophthalmic irAEs regardless of tumor type [6] (Table 2). Notably, uveitis developed in the present patient despite a prolonged remission period of 25 years, indicating that even a very remote history of uveitis may confer an increased risk of irAE-associated ocular inflammation. We did not initially recognize her remote history of uveitis as a potential risk factor and therefore did not perform a baseline ophthalmologic evaluation before treatment. Although mild ocular symptoms developed several days before the second dose of pembrolizumab, ophthalmologic evaluation was delayed until after the second dose, partly because we hesitated to interrupt neoadjuvant treatment given with curative intent. This delay may have contributed to treatment interruption, whereas earlier recognition of her high-risk status might have enabled earlier diagnosis.

**Table 2:** One-year incidence of ICI-associated uveitis in patients with pre-existing uveitis

Target	Agent	Braun D, et al. (Ref. 6)	Antoun J, et al. (Ref. 5)
		Patients with uveitis *n*/*N* (%)	Patients with uveitis *n*/*N* (%)
CTLA-4	Ipilimumab	8/16 (50.0%)	0/1 (0%)
PD-1	Nivolumab	39/99 (39.4%)	6/30 (20.0%)
PD-1	Pembrolizumab	44/100 (44.0%)	
PD-L1	Not reported	NA	0/7 (0%)
CTLA-4 /PD-1	Ipilimumab + Nivolumab	1/7 (14.3%)	1/3 (33.3%)

N, total number of patients with a history of uveitis; n, number of patients who developed uveitis after ICI initiation; CTLA-4, cytotoxic T-lymphocyte-associated antigen 4; PD-1, programmed cell death 1; PD-L1, programmed cell death ligand 1; NA, not available; ICI, immune checkpoint inhibitor

Current irAE management guidelines recommend topical corticosteroids for mild uveitis, discontinuation of ICIs with local and/or systemic corticosteroids for moderate uveitis, and high-dose systemic corticosteroids for severe cases [21–23]. Regarding outcomes, complete resolution occurred in 58% of patients, partial resolution in 25%, and recurrence despite treatment in 8% [8]. In the present case, uveitis responded promptly to low-dose systemic corticosteroids and resolved completely after steroid pulse therapy and intravenous immunoglobulin administered for incidentally developed SJS.

From an ophthalmologic perspective, immune-related uveitis appears to differ from conventional non-infectious uveitis in several important aspects. Previous studies have shown that ICI-associated uveitis often responds favorably to relatively low-dose corticosteroid therapy compared to typical autoimmune uveitis, suggesting a distinct inflammatory profile and generally good steroid sensitivity [24]. Additionally, uveitis may occur in the absence of overt structural retinal abnormalities on OCT, highlighting the importance of functional and clinical assessment beyond structural imaging alone [25]. Accordingly, functional testing, such as ERG, may aid in detecting subclinical retinal involvement and complement OCT in selected patients, as illustrated by bilateral ERG abnormalities in the present case despite unilateral structural changes on OCT. These findings indicate that ICI-associated uveitis may present with subtle or subclinical retinal involvement and may be effectively controlled with prompt corticosteroid therapy. Nevertheless, patients with high-risk features, including a prior history of uveitis or melanoma, require close ophthalmologic surveillance during ICI treatment, as early detection and intervention remain critical for preventing vision-threatening complications.

In clinical practice, TMP-SMX prophylaxis for *Pneumocystis jirovecii* pneumonia is recommended for patients receiving ≥ 20 mg/day of prednisolone for at least 4 weeks, particularly when combined with other immunosuppressive agents [26]. Although TMP-SMX is associated with a relatively high incidence of cutaneous adverse reactions, recent reports suggest that ICI-based therapy increases the risk of severe drug eruptions by lowering the threshold for drug hypersensitivity through immune activation [27, 28]. In retrospect, given the favorable response of uveitis to low-dose corticosteroids, prolonged steroid therapy and prophylactic TMP-SMX may have been unnecessary, and SJS might have been avoided. These findings highlight the importance of recognizing the potential risks associated with TMP-SMX use in patients receiving ICIs.

In the present case, pembrolizumab was discontinued after two cycles due to immune-related uveitis and SJS, leading to treatment interruption and reduced treatment intensity. Although marked tumor regression was observed, pCR was not achieved; therefore, postoperative treatment was completed with capecitabine based on the CREATE-X trial, which reported a 42% reduction in recurrence risk and a 48% improvement in OS among patients with residual disease [29]. In the event of disease recurrence, non-ICI-based therapies remain viable treatment options.

In conclusion, including the present case, previous reports have suggested that patients with a history of uveitis may be at increased risk of developing uveitis after ICI-based therapy. Careful pre-treatment evaluation, thorough patient counseling, and close multidisciplinary monitoring, with early involvement of ophthalmology specialists, are essential for the safe administration of ICIs in patients witha history of uveitis.

## Data Availability

The data supporting the findings of this case report are available from the corresponding author upon reasonable request.

## References

[1] Postow MA, Sidlow R, Hellmann MD (2018) Immune-Related Adverse Events Associated with Immune Checkpoint Blockade. N Engl J Med 378:158–168. 10.1056/NEJMra1703481 29320654 10.1056/NEJMra1703481

[2] Sumimoto H, Noda S, Koide H et al (2024) Pre-existing autoimmune disease as a risk factor for immune-related adverse events in cancer patients receiving immune checkpoint inhibitors. PLoS ONE 19:e0306995. 10.1371/journal.pone.0306995 39012903 10.1371/journal.pone.0306995 PMC11251620

[3] Lopez-Olivo MA, Kachira JJ, Abdel-Wahab N et al (2024) A systematic review and meta-analysis of observational studies and uncontrolled trials reporting on the use of checkpoint blockers in patients with cancer and pre-existing autoimmune disease. Eur J Cancer 207. 10.1016/j.ejca.2024.114148 10.1016/j.ejca.2024.114148 PMC11331889 38834015

[4] Antoun J, Titah C, Cochereau I (2016) Ocular and orbital side-effects of checkpoint inhibitors: a review article. Curr Opin Oncol 28:288–294. 10.1097/cco.0000000000000296 27136135 10.1097/CCO.0000000000000296

[5] Martens A, Schauwvlieghe PP, Madoe A et al (2023) Ocular adverse events associated with immune checkpoint inhibitors, a scoping review. J Ophthalmic Inflamm Infect 13:5. 10.1186/s12348-022-00321-2 36811715 10.1186/s12348-022-00321-2 PMC9947214

[6] Braun D, Getahun D, Chiu VY et al (2021) Population-Based Frequency of Ophthalmic Adverse Events in Melanoma, Other Cancers, and After Immune Checkpoint Inhibitor Treatment. Am J Ophthalmol 224:282–291. 10.1016/j.ajo.2020.12.013 33359682 10.1016/j.ajo.2020.12.013

[7] Sun MM, Kelly SP, Mylavarapu Bs AL et al (2021) Ophthalmic Immune-Related Adverse Events after Anti-CTLA-4 or PD-1 Therapy Recorded in the American Academy of Ophthalmology Intelligent Research in Sight Registry. Ophthalmology 128:910–919. 10.1016/j.ophtha.2020.11.001 33166553 10.1016/j.ophtha.2020.11.001

[8] Zhang HA, Yuan AT, Chiasson N et al (2025) Immune checkpoint inhibitor-associated Vogt-Koyanagi-Harada-like syndrome: A descriptive systematic review. J Ophthalmic Inflamm Infect 15:44. 10.1186/s12348-025-00484-8 40354015 10.1186/s12348-025-00484-8 PMC12069190

[9] Schmid P, Cortes J, Pusztai L et al (2020) Pembrolizumab for Early Triple-Negative Breast Cancer. N Engl J Med 382:810–821. 10.1056/NEJMoa1910549 32101663 10.1056/NEJMoa1910549

[10] Zhang H, Houadj L, Wu KY et al (2024) Diagnosing and Managing Uveitis Associated with Immune Checkpoint Inhibitors: A Review. Diagnostics 14:336. 10.3390/diagnostics14030336 38337852 10.3390/diagnostics14030336 PMC10855398

[11] Aisenbrey S, Lüke C, Ayertey HD et al (2003) Vogt-Koyanagi-Harada syndrome associated with cutaneous malignant melanoma: an 11-year follow-up. Graefes Arch Clin Exp Ophthalmol 241:996–999. 10.1007/s00417-003-0787-5 14618342 10.1007/s00417-003-0787-5

[12] Takeuchi M, Meguro A, Nakamura J et al (2023) HLA-DRB1*04:05 is involved in the development of Vogt–Koyanagi–Harada disease-like immune-related adverse events in patients receiving immune checkpoint inhibitors. Sci Rep 13:13580. 10.1038/s41598-023-40565-z 37604934 10.1038/s41598-023-40565-z PMC10442432

[13] Cortes J, Cescon DW, Rugo HS et al (2020) Pembrolizumab plus chemotherapy versus placebo plus chemotherapy for previously untreated locally recurrent inoperable or metastatic triple-negative breast cancer (KEYNOTE-355): a randomised, placebo-controlled, double-blind, phase 3 clinical trial. Lancet 396:1817–1828. 10.1016/s0140-6736(20)32531-9 33278935 10.1016/S0140-6736(20)32531-9

[14] Mittendorf EA, Zhang H, Barrios CH et al (2020) Neoadjuvant atezolizumab in combination with sequential nab-paclitaxel and anthracycline-based chemotherapy versus placebo and chemotherapy in patients with early-stage triple-negative breast cancer (IMpassion031): a randomised, double-blind, phase 3 trial. Lancet 396:1090–1100. 10.1016/s0140-6736(20)31953-x 32966830 10.1016/S0140-6736(20)31953-X

[15] Loi S, Salgado R, Curigliano G et al (2025) Neoadjuvant nivolumab and chemotherapy in early estrogen receptor-positive breast cancer: a randomized phase 3 trial. Nat Med 31:433–441. 10.1038/s41591-024-03414-8 39838118 10.1038/s41591-024-03414-8 PMC11835735

[16] Nederlof I, Isaeva OI, de Graaf M et al (2024) Neoadjuvant nivolumab or nivolumab plus ipilimumab in early-stage triple-negative breast cancer: a phase 2 adaptive trial. Nat Med 30:3223–3235. 10.1038/s41591-024-03249-3 39284953 10.1038/s41591-024-03249-3 PMC11564107

[17] Ignatiadis M, Bailey A, McArthur H et al (2025) Adjuvant Atezolizumab for Early Triple-Negative Breast Cancer: The ALEXANDRA/IMpassion030 Randomized Clinical Trial. JAMA 333:1150–1160. 10.1001/jama.2024.26886 39883436 10.1001/jama.2024.26886 PMC11783246

[18] Schmid P, Adams S, Rugo HS et al (2018) Atezolizumab and Nab-Paclitaxel in Advanced Triple-Negative Breast Cancer. N Engl J Med 379:2108–2121. 10.1056/NEJMoa1809615 30345906 10.1056/NEJMoa1809615

[19] Miles D, Gligorov J, André F et al (2021) Primary results from IMpassion131, a double-blind, placebo-controlled, randomised phase III trial of first-line paclitaxel with or without atezolizumab for unresectable locally advanced/metastatic triple-negative breast cancer. Ann Oncol 32:994–1004. 10.1016/j.annonc.2021.05.801 34219000 10.1016/j.annonc.2021.05.801

[20] Winer EP, Lipatov O, Im SA et al (2021) Pembrolizumab versus investigator-choice chemotherapy for metastatic triple-negative breast cancer (KEYNOTE-119): a randomised, open-label, phase 3 trial. Lancet Oncol 22:499–511. 10.1016/s1470-2045(20)30754-3 33676601 10.1016/S1470-2045(20)30754-3

[21] Haanen J, Obeid M, Spain L et al (2022) Management of toxicities from immunotherapy: ESMO Clinical Practice Guideline for diagnosis, treatment and follow-up. Ann Oncol 33:1217–1238. 10.1016/j.annonc.2022.10.001 36270461 10.1016/j.annonc.2022.10.001

[22] Schneider BJ, Naidoo J, Santomasso BD et al (2021) Management of Immune-Related Adverse Events in Patients Treated With Immune Checkpoint Inhibitor Therapy: ASCO Guideline Update. J Clin Oncol 39:4073–4126. 10.1200/jco.21.01440 34724392 10.1200/JCO.21.01440

[23] NCCN Clinical Practice Guidelines in Oncology (2026) Management of Immune Checkpoint Inhibitor-Related Toxicities (2026). vol Version 1

[24] Enomoto H, Kato K, Sugawara A et al (2021) Case with metastatic cutaneous malignant melanoma that developed Vogt-Koyanagi-Harada-like uveitis following pembrolizumab treatment. Doc Ophthalmol 142:353–360. 10.1007/s10633-020-09800-0 33392895 10.1007/s10633-020-09800-0

[25] Kato K, Nagashima R, Matsubara H et al (2025) Case of uveitis with increased electroretinographic amplitudes following Nivolumab and Ipilimumab administration for malignant melanoma. Doc Ophthalmol 150:183–188. 10.1007/s10633-025-10011-8 40072695 10.1007/s10633-025-10011-8

[26] Takeuchi K, Yakushijin Y (2021) Pneumocystis jirovecii Pneumonia Prophylaxis for Cancer Patients during Chemotherapy. Pathogens 10:237. 10.3390/pathogens10020237 33669742 10.3390/pathogens10020237 PMC7923143

[27] Urasaki T, Ono M, Mochizuki T et al (2021) Case Report: A Case of Trimethoprim/Sulfamethoxazole-Triggered Hypotensive Shock: Cytokine Release Syndrome Related to Immune Checkpoint Inhibitors and Drug-Induced Hypersensitivity Syndrome. Frontiers in Oncology 11–2021. 10.3389/fonc.2021.681997 10.3389/fonc.2021.681997 PMC8121494 33996612

[28] Pavlos R, White KD, Wanjalla C et al (2017) Severe Delayed Drug Reactions: Role of Genetics and Viral Infections. Immunol Allergy Clin North Am 37:785–815. 10.1016/j.iac.2017.07.007 28965641 10.1016/j.iac.2017.07.007 PMC5702581

[29] Masuda N, Lee S-J, Ohtani S et al (2017) Adjuvant Capecitabine for Breast Cancer after Preoperative Chemotherapy. N Engl J Med 376:2147–2159. 10.1056/NEJMoa1612645 28564564 10.1056/NEJMoa1612645

